# Acoustic probing of the particle concentration in turbulent granular suspensions in air

**DOI:** 10.1038/s41598-020-73427-z

**Published:** 2020-10-06

**Authors:** S. van den Wildenberg, X. Jia, O. Roche

**Affiliations:** 1 Université Clermont Auvergne, CNRS, IRD, OPGC, Laboratoire Magmas et Volcans, 63000 Clermont-Ferrand, France; 2 Institut Langevin, ESPCI Paris, PSL University, CNRS, 75005 Paris, France

**Keywords:** Acoustics, Fluid dynamics, Volcanology, Techniques and instrumentation

## Abstract

Dilute gas–particle suspensions in which the particles are carried by the fluid are found in various industrial and geophysical contexts. One fundamental issue that limits our understanding of such systems is the difficulty to obtain information on the particle concentration inside these often optically opaque suspensions. To overcome this difficulty, we develop ultrasonic spectroscopy to monitor the local particle concentration $\phi$ of glass particles (with diameters $d\sim$ 77 $\upmu$m or 155 $\upmu$m) suspended in air. First, we determine the minimal air velocity, $U^*$, necessary to suspend the particles from the maximum decrease in the transmitted wave amplitude and velocity of ultrasound propagating through the suspension. Next, setting the air velocity at $U^*$, we increase the mass of particles and monitor acoustically the local solid volume fraction, $\phi$, by measuring the ultrasound wave attenuation coefficient and phase velocity as a function of frequency on the basis of classical scattering and hydrodynamic models. For the frequency ranges and suspensions considered here, the viscous dissipation dominates over scattering and thermal conduction losses. We show that, for a characteristic air velocity $U^*$, the locally measured $\phi$ reaches a critical value, in agreement with a recent study on turbulent gas–particle mixtures. Moreover, we find that this critical $\phi$ increases with the size of the particles. Finally, analysis of the temporal fluctuations of the locally measured solid volume fraction, suggests that high density regions (clusters) are present even in suspensions with concentrations below the critical concentration. This differs from the current hypothesis according to which the critical concentration coincides with the onset of cluster formation.

## Introduction

Dilute mixtures of particles in a gas are common in industry and in nature. Examples include dust storms^1^, snow surge avalanches^2,3^, and pyroclastic density currents^4–7^. The size of the particles in these gas–particle mixtures varies greatly, but a typical average particle size is about $100\; \upmu \hbox {m}$^6,8^. The mechanical properties and dynamics of these mixtures is, to a large extent, determined by their solid volume fraction defined as the ratio of the volume occupied by the solid particles with respect to the total suspension volume. The solid volume fraction affects the fluid turbulence^9^, and controls the degree of coupling between the gas and the particles as well as the intensity and the frequency of the particle collisions^10^. Particle image velocimetry (PIV) has greatly contributed to advances in the understanding of turbulent and complex flows in dilute suspensions (typically below $\phi <10^{-5}$) of fine particles (typically $\sim$ 1–10 $\upmu$m)^11,12^. One major issue that limits our understanding of turbulent gas–particle suspensions is the difficulty to determine the solid volume fraction inside these mixtures which are often opaque. Indeed, the relatively large size and high concentration ($\sim$ 1–10%) of the particles complicate optical observation inside such suspension due to the extensive light scattering by the particles. One approach to avoid this difficulty is to determine the fluid pressure using pressure sensors^6,8^. The bulk solid volume fraction can be calculated from the fluid pressure, the height of the suspension above the sensor and the important assumption that the density of the mixture is homogeneous. This assumption is probably not correct as the height of the suspension increases and heterogeneities become important. Another approach is based on image analysis to establish an empirical relationship between the local grey scale and the local solid volume fraction^7^. However, this relationship has to be validated by separate intrusive measurements, making the approach tedious to use in many real 3D systems. Despite their limitations, these approaches have shown that the solid volume fractions of turbulent gas–particles mixtures are typically between 0.1 and 10%^5,6^. However, a more adequate method to determine the solid volume fraction is desirable.

In this study we introduce a non intrusive approach called acoustic probing, which is based on ultrasound spectroscopy. Ultrasound spectroscopy has been applied on a wide variety of materials in which particles or droplets are suspended in an aqueous phase. The principle relies on measurements of the velocity and attenuation of an ultrasound signal propagating through the system. The interaction of ultrasound with the material depends on the contrast between the constituent components, the size distribution and the concentration of the particles. There exists a substantial number of models relating the sound speed and attenuation to the physical properties of the system^13,14^. For example, ultrasound spectroscopy has been applied to obtain: the bulk modulus of particles^15^, the effective porosity of porous particles^16^, the solid volume fraction of dilute suspensions of ice in water^17^, the effect of particle charge in moderately concentrated suspension^18^.

We use acoustic probing for the first time, to the best of our knowledge, to monitor the local solid volume fraction inside optically opaque turbulent gas–particle mixtures (dilute suspensions). We start by suspending a mass of non-Brownian glass beads by increasing the upward velocity of the air. The minimum flow velocity necessary to suspend the particles, $U^*$, is determined from the strongest sound-suspension interaction when the solid particles cross the probing ultrasonic beam. Then, the local solid volume fraction is inferred by ultrasound velocity and attenuation measurements using appropriate wave scattering and hydrodynamic models. Finally, we discuss two important properties of these mixtures, namely, the maximum solid volume fraction $\sim$ 5–10% that may be suspended at $U^*$, and the formation of particle clusters.

## Methods

### Setup

**Figure 1:**
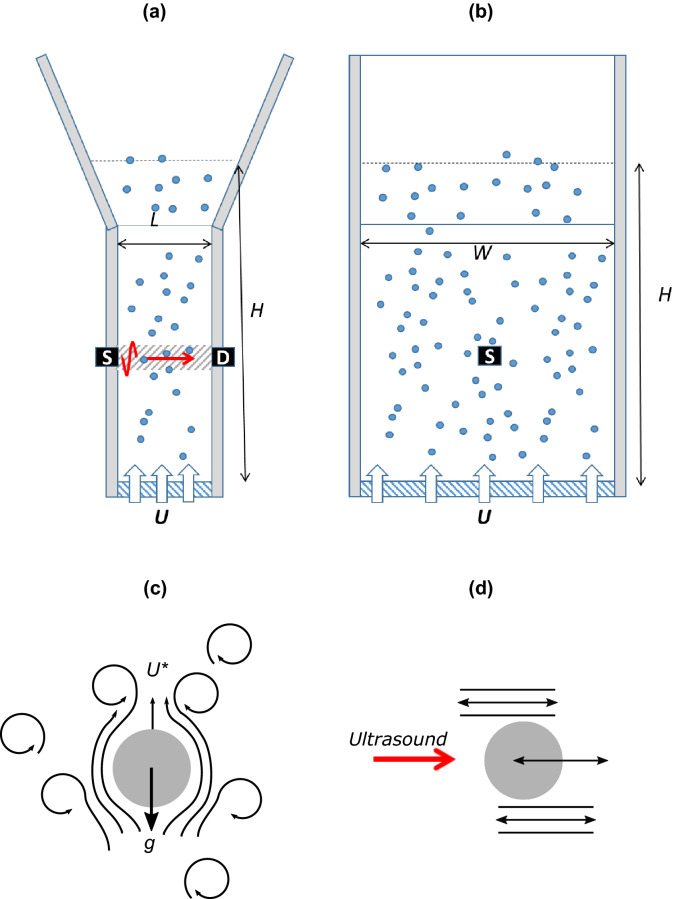
Schematic of the setup, (**a**) front view, (**b**) side view. The setup consists of a rectangular plexiglass container with a width (*W*) of 15 cm, a length (*L*) of 2 cm and a porous plate at the bottom (dashed). A suspension of glass beads in air is obtained by supplying an air flow at a velocity *U* via the porous plate. The height (*H*) of the sample is set by *U* and is about 21 cm. Ultrasonic waves are generated by a source transducer (S) on one side of the container and the propagating wave is measured by a second transducer (D). The volume probed by the acoustic wave, corresponding to the transducer positions, is indicated by the grey dashed area. (**c**) Cartoon of the minimal air velocity $U^*$ required to suspend the particles, the flow regime being defined at two different length scales via $\hbox {Re}_p$ at the scale of the particle and $\hbox {Re}_{{mix}}$ at the scale of the container. (**d**) Cartoon of the oscillating motion of the denser particle with respect to less dense continuous phase, induced by the incident acoustic wave (red arrow). The relative motion results in frictional losses, which are commonly modelled by viscous (shear) waves^14^.

The setup consists of a plexiglass container with a width *W* = 15 cm and a length *L* = 2 cm (Fig. 1a,b). The long walls of the container diverge at the top to allow the air velocity to decrease to zero and avoid loss of particles without the use of a grid. Indeed, a grid may cause an increase in fluid pressure as air flow is hampered by the grid and by the particles that are trapped in the interstices of the grid. The disadvantage of not using a grid is the error in the observation of *H*. However, we performed four independent experiments and we estimated the spread between these experiments to be greater than the error due to variations in *H*. The container is connected to an air supply system at the base, which delivers mean air velocities up to nearly 1 m/s through a porous plate at the bottom. The particles are glass beads with an average diameter (*d*) of 77 $\upmu$m or 155 $\upmu$m. The glass beads have a narrow size distribution, having been sieved to limit *d* to a narrow range between 75 and 80 $\upmu$m and between 150 and 160 $\upmu$m, respectively. An experiment is carried out as follows. A known mass of glass beads is poured into the container and subsequently suspended by an upward airflow *U* to counterpart the particle settling velocity.

The nominal input solid volume fraction $\phi _i=m_g/(\rho _g V_t)$ is calculated, assuming a homogeneous suspension, from the mass of poured glass beads ($m_g$), the density of the glass beads ($\rho _{g}$ = 2500 kg/$\hbox {m}^3$) and the total volume occupied by the gas–particle mixture ($V_t$). The $V_t$ is calculated from the dimensions of the container and the height of the suspension at the minimal air flow velocity $U^{*}$ (see below). We estimate an error in the measurement of *H* of about 1 cm, and using error propagation we calculate the horizontal errorbars in the input solid volume fraction shown in Fig. 5. The nominal input solid volume fraction is then gradually varied from 0.1 to 10 % by pouring more glass beads, $m_g$, into the container (with the air velocity stopped). For a given velocity $U^*$, we do not observe an appreciable change in the height (i.e. $V_t$) between the different input solid volume fractions tested in this study. For each $\phi _i$ the propagation of about 400 to 500 ultrasonic pulses is measured and the mean attenuation coefficient and mean phase velocity are determined by averaging over these pulses. The air flow is stopped and $\phi _i$ is varied up to 10 % by pouring in more particles. The experiment is repeated 4 times.

### Estimation of the mixture flow regime

To characterize the mixture flow in our experiments, we consider the Reynolds number of the mixture $Re_{mix} = \rho _{mix} U D/ \eta$. Here $\rho _{mix}= \phi \rho _{g}+(1-\phi ) \rho _{air}$, $\rho _{air}$ = 1.2 kg/$\hbox {m}^3$, *U* is the mean air velocity, $\eta$ is the dynamic air viscosity and *D* is a typical length scale of the container. This shows that the $Re_{mix}$ increases with the solid volume fraction of the suspension. For our rectangular container, in the experiments with the *d* = 77 $\upmu$m glass beads, taking *U*= 0.4 m/s and volume fractions $\phi$ from 0.005 to 0.09, this yields a range of values for $Re_{mix}$: (i) in the direction *L*, $Re_{mix}\sim$ 6$e^3-1e^5$, and (ii) in the direction *W*, $Re_{mix}\sim$ 4$e^4-7e^5$. For the experiments with the larger beads, *d* = 155 $\upmu$m and *U*= 0.9 m/s this yields a $Re_{mix}\sim$ 1$e^4-2e^5$ , and $Re_{mix}\sim$ 1$e^5-1e^6$, respectively. In general, for flows through a pipe, flows are considered laminar at $Re<$ 2000, fully turbulent at $Re>$ 4000, and in the transitional regime for *Re* in between. This suggests that, even though the flow in our rectangular container may be complex, the experiments in this study are probably conducted in the turbulent flow regime.

### Ultrasonic measurements

Ultrasonic waves are generated by a source piezo electric transducer (S) on one side of the container and the propagating wave is measured at a distance *L* by a similar second transducer (D) (Fig. 1a,b). The transducers have a nominal element size of 6 mm. As the glass beads and the air have a large mismatch in the acoustic impedance $Z=\rho c$, with $\rho$ the material densities and *c* the sound velocities, we may expect significant scattering. In our experiments the central frequency of ultrasound *f* = 0.5MHz, corresponding to a wavelength about $\lambda =c_{air}/f$ = 0.7 mm (with $c_{air}$ = 350 m/s). Since the surface diameter of the detecting transducers is about 10 times larger than the characteristic size of the ultrasound speckles of the order of $\lambda$, more than a dozen speckles are averaged over the detector transducer. Therefore, we obtain good cancellation of the scattering wave while leaving the spatially coherent ballistic pulse unaffected.

For a given $\phi _i$, we send 500 short pulses, so that the content of each pulse extents over many frequencies, with a repetition time of 30 ms. The particles are in continuous motion, moving a mean distance that is negligibly small over the time the pulse takes to travel through the sample. However, the motion of the particles is significant, with respect to the wavelength, over the time between pulses. Thus for a given $\phi _i$, we measure many different ensembles of the particles.

Determination of the solid volume fraction of a granular suspension relies on being able to accurately measure the frequency dependence the attenuation coefficient $\alpha (f)$ and/or the phase velocities $v_{\theta }(f)$. For a given $\phi _i$, we take for each pulse only the coherent part of the transmitted signal and determine $\alpha (f)$ and $v_{\theta }(f)$ from the fast Fourier transform (FFT) using the relations:

1 $$\begin{aligned} \alpha (f)= & {} -\frac{1}{L}ln\frac{A_s}{A_{air}}, \end{aligned}$$

2 $$\begin{aligned} v_\theta (f)= & {} \frac{2 \pi f L}{(\theta _s-\theta _{air})+\frac{2 \pi f L}{v_{air}}+2 m \pi }, \end{aligned}$$

where *L* is the sample size, *A*(*f*) and $\theta (f)$ are the amplitude and phase from the FFT. Furthermore, the subscript ‘*s*’ refers to the suspension, and the factor 2m$\pi$ is to unwrap the phase. The attenuation coefficient and phase velocity spectra were averaged over all 500 pulses to obtain a single attenuation and velocity spectrum for each $\phi _i$.

### Ultrasound attenuation and velocity in two-phase suspensions

In this study, the solid volume fractions tested are less than 10$\%$. The ultrasonic spectroscopy developed for dilute aqueous suspension^19^ could thus be applied to our gas–particle mixtures (i.e., two-phase suspensions). This is supported by the fact that in the steady state the mixture flow velocity $U\sim$ 1 m/s is much less than than the sound wave velocity $c\sim$ 350 m/s. We identify two approaches to study sound propagation through solid-in-liquid suspensions, namely: (i) the coupled-phase hydrodynamic model in the very long wavelength limit ($\lambda /d\gg$ 1)^15,20–22^, and (ii) the scattering model on the microscope scale ($\lambda /d\sim$ 1)^23,24^. Below we give a brief description of these two approaches.

Urick^21^ considers a system with a volume fraction $\phi$ of solid particles with density $\rho _2$ and compressibility $\kappa _2$, suspended in a continuous phase with density $\rho _1$ and compressibility $\kappa _1$. To determine the acoustic loss we will use the model developed by Urick, who derived the ultrasound attenuation in such system from the $\phi$ and an ‘average’ scattering factor^21^. More precisely, Urick followed the early work of Lamb where the sound attenuation was related to the scattering from a small rigid sphere suspended in a viscous continuous phase, and approximated the attenuation coefficient in a dilute suspension as^21^:

3 $$\begin{aligned} \alpha =\phi \left[ \frac{1}{6} k^4 a^3+k(\sigma -1)^2 \frac{s}{s^2+(\sigma +\tau )^2)} \right] , \end{aligned}$$

where *a* is the particle radius, the wavenumber $k=\omega /v$ with $\omega$ the angular frequency, and $\sigma =\rho _2/\rho _1$, $\tau =(1/2)+(9/4)(\delta / a)$ and $s=(9/4 )[(\delta /a)+(\delta /a)^2]$. Here $\delta =\sqrt{2\eta /(\omega \rho _1)}$ is a characteristic viscous (shear wave) length, with $\eta$ the dynamic viscosity of the continuous phase. The first of the two terms in Eq. (3) is the Rayleigh scattering loss produced by a small rigid sphere free to move, and the second term represents a viscous loss as the suspended particle oscillates with respect to the surrounding fluid in the sound field (Fig. 1d). In the frequency range that we are investigating ($\sim$ 1 MHz) and the high density contrast ($\sigma \gg$1) the visco-inertial loss is larger than the scattering loss. Moreover, by computing the viscous drag force exerted by the fluid on the sphere, given by Stokes law in the long wavelength limit, Urick derived the rate of energy loss and an absorption coefficient identical to the second term in Eq. (3).

This hydrodynamic approach can also be adopted to calculate the sound velocity *v* in the suspension. The idea was to assume an effective density $\rho _{eff}$ and an effective compressibility $\kappa _{eff}$ of the mixture by taking a volume weighted average of the densities and the compressibilities of the two phases, with *v* found from Wood equation:

4 $$\begin{aligned} v=(\rho _{eff} \kappa _{eff})^{-1/2}. \end{aligned}$$

The simplest assumption is that^15^ $\rho _{eff}=\phi \rho _2+(1-\phi )\rho _1$ and $\kappa _{eff}=\phi \kappa _2+(1-\phi )\kappa _1$. Improvements to the averaging of the density were made by Ament^20^, who included the coupling effects between the two phases as a function of inertia, viscosity and particle size. For $\delta /a\ll$1 the suspension is in the inertial regime, while for $\delta /a\gg$1 it is in the Stokes regime^16^. Here we have $\delta /a \sim$ 0.03 pertaining to the inertial regime. The effective density is then given by^13,20^:

5 $$\begin{aligned} \rho _{eff}= \phi \rho _2 + (1-\phi )\rho _1-2(\rho _2-\rho _1)^2\phi (1-\phi )\frac{Q}{Q^2+U^2}, \end{aligned}$$

where $Q=2(\rho _2-\rho _1)(1-\phi )+(9/2)(\delta /a)\rho _1+3\rho _1$ and $U=(9/2)\rho _1[\delta /a+(\delta /a)^2]$. In this approach, which we refer to as Urick/Ament, $\phi$ is obtained by fitting the experimental data using Matlab’s least-squares curve fitting tools. The experimental attenuation is fitted by Eq. (3) including an offset (see section “Discussion” in main text), and the velocity data is fitted by Eq. (4) where $\rho _{eff}$ is given by Eq. (5). Within the hydrodynamic approach, Harker and Temple^22^ developed a more general model to the derivation of the effective density and compressibility for calculating the complex propagation constant (wavenumber), applicable to more concentrated particle suspension. However, we find this model does not provide better agreement with our data compared to the predictions by Urick/Ament.

The second approach is based on scattering models developed by Epstein and Carhart^24^, Allegra and Hawley^23^ referred to as the ECAH theory^14,19^. The ECAH theory describes the interactions of scattered, viscoinertial, and thermal fields with a single particle and its surrounding medium^19,23–25^. The frequency dependent $\alpha$ and *v* are calculated from the effective complex wavenumber, $K=\omega /v+i\alpha$. In this study we use a comprehensive model based on multiple scattering theory^26^:

6 $$\begin{aligned} K^2=k_1^2+\frac{3\phi }{a^3}f(0)+\frac{9 \phi ^2}{4 k_1^2 a^6} \left[ f^2(0)-f^2(\pi )\right] . \end{aligned}$$

Here $k_1$ is the complex wavenumber in the continuous phase $k_1=\omega /v_1+i\alpha _1$, where $v_1$ and $\alpha _1$ are the sound velocity and absorption coefficient, respectively. Furthermore, *f*(0) and $f(\pi )$ give the forward and backward scattering amplitudes of the individual particles:

7 $$\begin{aligned} f(0)= & {} \frac{1}{ik_1}\sum \limits _{n=0}^{\infty }(2n+1)A_n, \end{aligned}$$

8 $$\begin{aligned} f(\pi )= & {} \frac{1}{ik_1}\sum \limits _{n=0}^{\infty }(-1)^n(2n+1)A_n. \end{aligned}$$

The $A_n$ terms are the scattering coefficients of the various types of waves scattered from the individual particles. A rigorous approach to calculate the scattering coefficients for spherical objects was developed by Epstein and Carhart^24^ for liquid/liquid systems and Allegra and Hawley for solid/liquid systems^23^. Equivalence between approaches, including the model for solid/solid systems^25^, was established by introducing ‘wild card’ variables^19^. The ECAH model allows the calculation of the $A_n$ terms, for each *n*, by solving a series of 6 $\times$ 6 complex simultaneous equations. For detailed explanations on the ECAH model we refer to references^14,19,27^. If the thermal contribution is neglected (reasonable if the density contrast between the phases is high) the computation reduces to 4 $\times$ 4 set of equations, which are given in Challis et al.^19^. To obtain $\phi$ from the experimental data, the theoretical $\alpha$ and *v* are compared to the experiments using Matlab’s least-squares curve fitting tools.

As we will show later, the predictions by both Urick/Ament and ECAH models agree fairly well in our experimental ranges, in particular for the attenuation coefficient. Indeed, the $A_0$ and $A_1$ terms in Eqs. (7) and (8) represent the monopole and dipole scattering, which corresponds precisely to the first and second terms in Eq. (3). Certainly in solid-in-liquid suspensions considered here at low solid volume fractions and with relatively small particles compared to the wavelength, Urick/Ament model for both velocity and attenuation are simpler to apply and may be no less accurate in simulating measured results.

## Results

We investigate the ballistic propagation of ultrasound in dilute suspensions of glass beads in air. We first measure the air flow velocity necessary to suspend glass beads of diameter *d* = $77\;\upmu \hbox {m}$. To do so, we pour a known volume of beads into the container and step-wise increase the upwards air flow velocity *U* (Fig. 1a,b). At each air flow velocity, 400 short pulses are send by a piezo-electric transducer and the propagating pulses are measured at a distance $L\sim$ 0.02 m using a second transducers.

**Figure 2:**
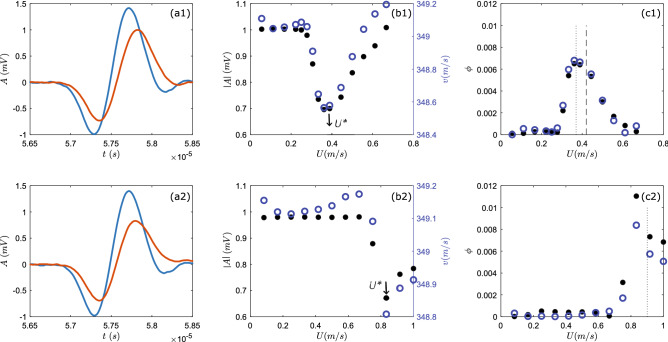
(**a1**) Example of a transmitted ultrasound pulse measured in a suspension of glass beads ($\phi _i\sim$0.006) in air, at an air flow velocity *U*= 0.22 m/s (in blue) and *U*= 0.44 m/s (in orange) using glass beads *d* = 77 $\upmu$m. (**a2**) Example of a transmitted ultrasound pulse measured at *U* = 0.5 m/s (in blue) and *U* = 0.84 m/s (in orange) using glass beads *d* = 155 $\upmu$m. (**b1**) The mean absolute amplitude (dots) and the mean time-of-flight velocity (circles) of ultrasound pulses as a function of *U*, for glass beads *d* = 77 $\upmu$m. (**b2**) Same as (**b1**) for glass beads *d* = 155 $\upmu$m. (**c1**) The packing fraction of a suspension of glass beads *d* = 77 $\upmu$m in air as a function of *U*, obtained from fitting the attenuation coefficient (dots) and phase velocity (circles) using Urick/Ament models (see text). (**c2**) Same as (**c1**) for glass beads *d* = 155 $\upmu$m.

Examples of ultrasonic pulses propagating through a suspension at low and high *U* reveal a retardation and a decrease of the pulse amplitude (Fig. 2a1). For each *U*, the amplitude *A* and the time-of-flight velocity *v* of the pulse are obtained from the arrival of the first minimum and averaged over the 400 pulses (Fig. 2b1). For *d* = 77 $\upmu$m and low *U*, *A* and *v* remain nearly constant till $U\sim$ 0.25 m/s after which they rapidly decrease. For $U>$ 0.45 m/s, *A* and *v* steadily increase again, indicating that for sufficiently large *U* the particles are dragged above the position of the transducers; we will not further investigate this flow regime. Experiments conducted with glass beads of *d* = 155 $\upmu$m, show a similar behavior, however, *A* and *v* start to decrease at a significantly higher air velocity $U\sim$ 0.8 m/s (Fig. 2a2,b2).

We define $U^*$ as the air flow velocity at which the *A* and *v* of the propagating ultrasound pulse are lowest, and the effect of the sound-particles interaction is thus maximal. For the beads of *d* = $77\;\upmu \hbox {m}$ this corresponds to $U^*\sim$ 0.4 m/s, while for the *d* = $155\;\upmu \hbox {m}$ beads this gives $U^*\sim$ 0.9 m/s. These air flow velocities are used in the experiments hereafter. The nominal solid volume fraction $\phi _i$ is obtained from the total volume occupied by the suspension at $U^*$. It is interesting to compare the above velocity $U^*$ to the terminal velocity $U_t$ of a solid particle falling in a single-phase fluid (Fig. 1c), calculated with the Stokes law in a creep (laminar) flow regime or with the method described in Rhodes^28^ for the intermediate flow regime. For glass beads of diameter $d= 77 \ \upmu \mathrm{m}$ the particle Reynolds number $Re_p=\rho U^* d/\eta \sim$2. Stokes law gives $U_t=d^2g\Delta \rho /(18\eta )\sim$ 0.42 m/s, while Rhodes method gives $U_t\sim$ 0.38. Both these values correspond fairly well with $U^*$. Instead, for glass beads of size *d* = $155\;\upmu \hbox {m}$, stokes law gives us $Ut\sim$ 1.8 m/s while Rhodes method yields $U_t\sim$ 0.9 m/s. This indicates that for these larger particles the assumption of creep flow fails and we find that $U^*$ is closer to $U_t$ predicted by Rhodes method. Indeed, $Re_p\sim$ 10 indicating that the flow regime at the particle scale is in the intermediate turbulent regime^28^. At larger length scales the flow of the suspension is set by the $\hbox {Re}_{{mix}}$ (see “Methods” section).

**Figure 3:**
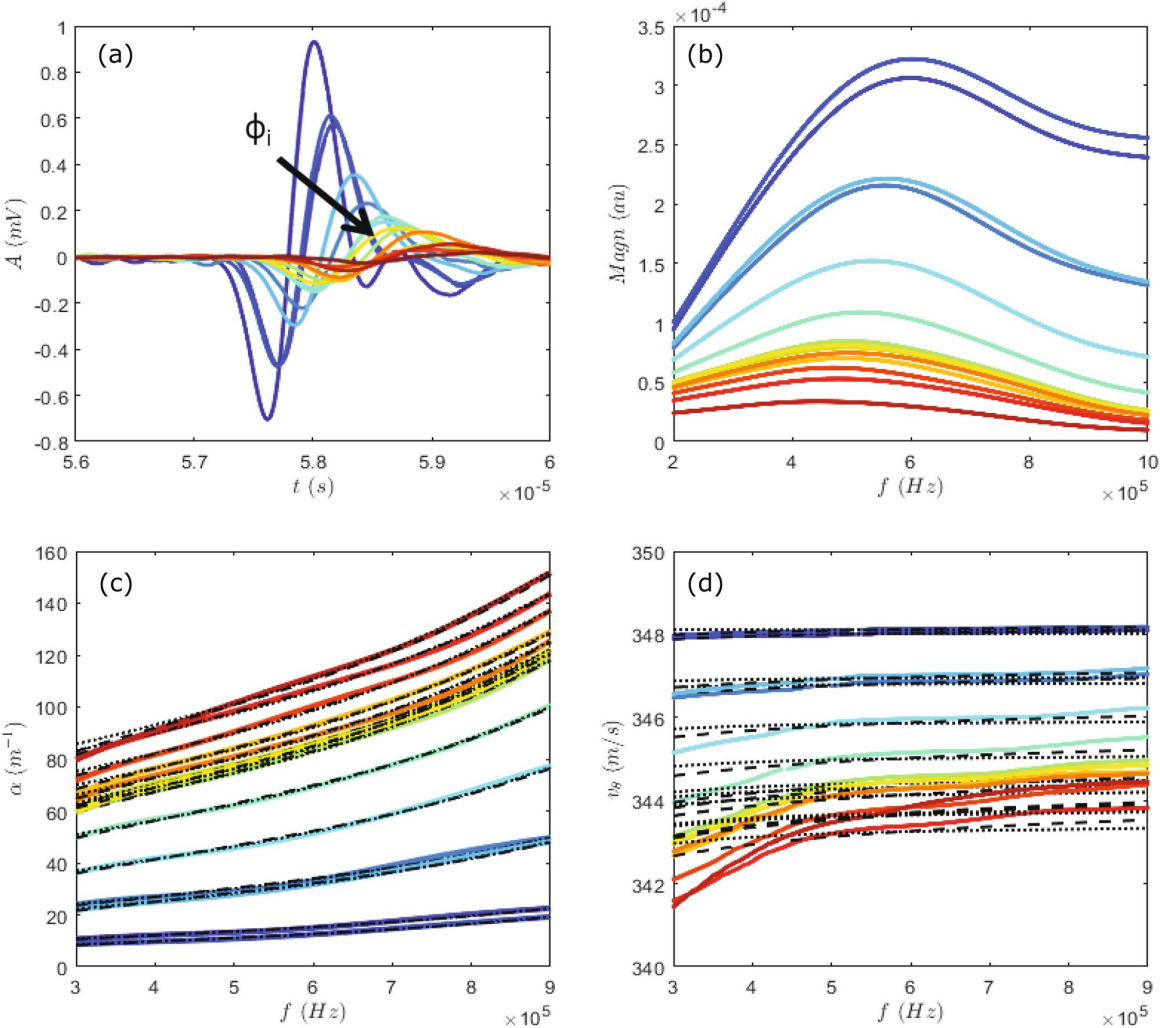
Typical ultrasound experiment in a suspension of glass beads $d=$ 77 $\upmu$m in air. (**a**) Examples of measured acoustic pulses for increasing particle concentrations (black arrow). For each concentration, 400 of such pulses were measured. (**b**) Fast Fourier transform of the pulses shown in (**a**). (**c**) Mean attenuation coefficient as a function of frequency for different $\phi _i$. (**d**) Mean phase velocity as a function of frequency $\phi _i$. Black dotted lines are the fits by ECAH44 model, and dashed black lines are fits by Urick/Ament model.

**Figure 4:**
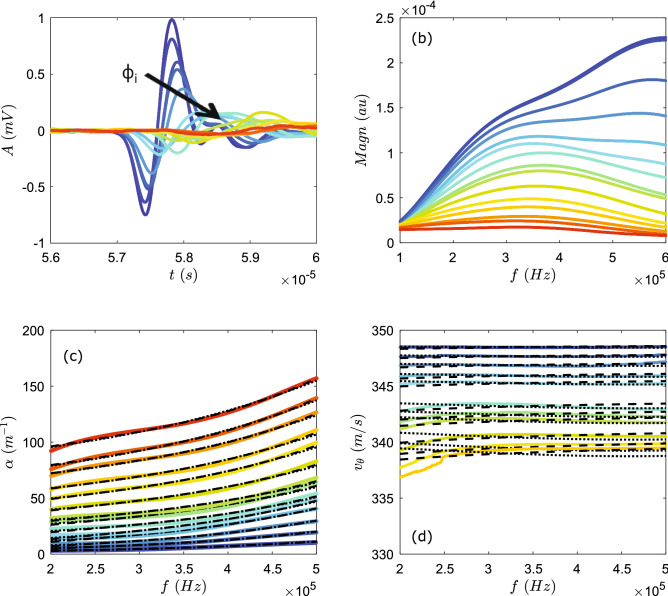
Typical ultrasound experiment in a suspension of glass beads $d=$ 155 $\upmu$m in air. (**a**) Examples of measured acoustic pulses for increasing particle concentrations (black arrow). For each concentration, 400 of such pulses were measured. (**b**) Fast Fourier transform of the pulses shown in (**a**). (**c**) Mean attenuation coefficient as a function of frequency for different $\phi _i$. (**d**) Mean phase velocity as a function of frequency $\phi _i$. Black dotted lines are the fits by ECAH44 model, and dashed black lines are fits by Urick/Ament model.

Next, the dependence of the ultrasound propagation on the solid volume fraction of the suspension is investigated. For this experiment, a known mass of glass beads *d* = 77 $\upmu$m is poured into the container and suspended using $U^*$ = 0.4 m/s. The nominal solid volume fraction of the suspension is incremented from 0–10$\%$ by adding more beads into the container. As mentioned above, we do not observe a significant change in height for different suspension tested here, indicating that the occupied volume of these mixtures appears independent on the solid particle concentration. The propagating ultrasound pulses measured in suspensions for increasing $\phi _i$ display a decrease of the amplitude and the time-of-flight velocity (Fig. 3a). To investigate in detail the frequency dependence of the ultrasound scattering a spectral analysis is performed. The spectra evidence that the center frequency of the propagating pulses decreases from 0.6 MHz for $\phi _i\sim$ 0.01 to nearly 0.4 MHz for $\phi _i\sim$ 0.1 (Fig. 3b). For each $\phi _i$, the frequency dependent $\alpha$ and $v_{\theta }$ are calculated from the spectra via Eqs. (1) and (2), respectively, and averaged over all the pulses (Fig. 3b,c). We focus on the frequency range between 0.3 and 0.9 MHz where $\alpha$ and $v_{\theta }$ appear significant for all $\phi _i$. In this frequency range, we find that there is a more important increase for $\alpha$ than for $v_{\theta }$ with increasing frequency. Instead, for increasing $\phi _i$, $\alpha$ increases but $v_{\theta }$ decreases.

The experiments are repeated with suspension of $d\sim$ 155 $\upmu$m glass particles and $U^*$= 0.9 m/s. The propagating ultrasound pulses exhibit a similar behavior as for the smaller beads, i.e. a decrease of the ultrasound amplitude and velocity with increasing $\phi _i$ (Fig. 4). The fast Fourier transform reveals, however, that the center frequency of the propagating pulse is significantly lower than for the smaller beads. This is expected as the particles act as a filter where the cut off frequency is related to the size of the particles. Therefore, $\alpha$ and $v_{\theta }$ are now evaluated in the lower and narrower frequency range between 0.2–0.5 MHz. Within this range we observe, first, a slight dispersion of $\alpha$ and $v_{\theta }$ and second, for increasing $\phi _i$, the increase of $\alpha$ and decrease of $v_{\theta }$.

## Discussion

The light scattering by the grains inside a 3D granular suspension makes it difficult to optically determine the solid volume fraction. Therefore, we monitor the local solid volume fraction of a suspension of micro-meter sized glass beads in air via acoustic probing. We find that the acoustic attenuation coefficient and velocity decrease with increasing input solid volume fraction. To obtain the local solid volume fraction of the suspension, we now analyse the frequency dependence of the measured $\alpha$ and $v_{\theta }$ using both hydrodynamic and scattering models.

Before embarking on a more detailed analysis, it is useful to first consider the wave scattering regime in which we are working. To this end, we evaluate the product of the wavenumber and the particle radius $ka=(\pi d f)/c_{air}$. In our experiments taking $f\sim$ 0.5 MHz gives $ka\sim$ 0.3 for the *d* = 77 $\upmu$m beads and $ka\sim$ 0.7 for the *d* = $155\;\upmu \hbox {m}$ beads. Both these values correspond to the long to intermediate wave scattering regime ($ka<$1), where (isotropic) scattering might not be negligible. Additionally, the density contrast between the air and the solid phase is high, thus we may expect that viscous or inertial interactions are important (Fig. 3b). With these considerations, we focus specifically on the models: Urick/Ament and ECAH44 (see “Methods” for details). In the Urick/Ament model, the velocity data are fitted by Eq. (4) and the attenuation data by $\alpha =\zeta +\alpha _{Eq.3}$. In the ECAH44 model, the velocity and attenuation data are fitted by $v_{\theta }=\omega /Re[K_{Eq.6}]$ and $\alpha =\zeta +Im[K_{Eq.6}]$, respectively. Hence, $\alpha (f)$ is fitted using the free fitting parameters $\phi$ and $\zeta$. Here $\zeta$ represents an additional fit parameter that accounts for a frequency-independent offset but depends on the solid volume fraction $\phi _i$. We find that $\zeta$ varies from about 1 $\hbox {m}^{-1}$ at low $\phi _i$ ($\sim$ 0.01) to 30 $\hbox {m}^{-1}$ at high $\phi _i$ ($\sim$ 0.1), corresponding to a correction on the measured acoustic attenuation from 2 to 45%. As our ultrasonic attenuation is determined by the ratio of the transmitted wave amplitude through the gas–particle mixture and that through air (via Eq. 1), this additional loss associated with $\zeta$ could arise from the reflection between air (considered as wave incidence medium) and the mixture (sample) due to the impedance mismatch: as expected, the higher is the volume fraction, the larger is the impedance mismatch and consequently the additional loss. To better quantify such effect, we need to further investigate this issue on the basis on the reflection/transmission of a fluid-borne wave through a diphasic suspension (or porous medium)^29^. Note finally that there is good agreement between the solid volume fractions inferred from the attenuation data and the velocity data in which such extra fitting parameter ($\zeta$) is not necessary, because the reflection/transmission would not affect our velocity measurement.

**Figure 5:**
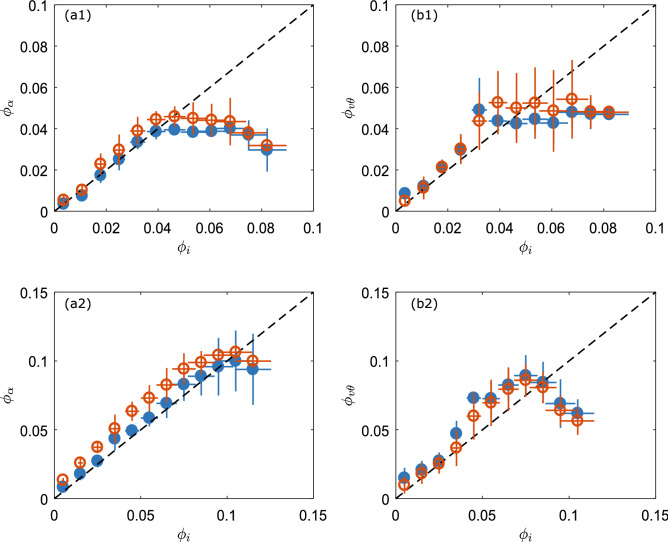
Locally measured volume fractions as a function of nominal solid volume fraction. For the suspension of 77 $\upmu$m beads, (**a1**) $\phi _{\alpha }$ obtained from fitting the experimental $\alpha (f)$, and (**b1**) $\phi _v{\theta }$ obtained from fitting the experimental $v_{\theta }(f)$, with the Urick/Ament model (blue solid symbols) and the ECAH44 model (orange open symbols). (**a2**) and (**b2**) same for the suspension of 155 $\upmu$m beads. The data of 4 experiments are binned and vertical errorbars represent the standard error of the mean for each bin. The errors in $\phi _i$ due to uncertainty in the height measurement (see “Methods” section) are represented by the horizontal errorbars.

These models describe well the experimentally obtained ${\alpha }$ and ${v_{\theta }}$ in suspension of 77 $\upmu$m beads (black lines in Fig. 3c,d). The different models yield similar local solid volume fractions, which are presented as a function of the $\phi _i$ in Fig. 5a1,b1. The two main features are the following. (i) for $\phi _i<$ 4%, $\phi _\alpha$ and $\phi _v$ are comparable to $\phi _i$. (ii) for $\phi _i>$ 4%, $\phi _\alpha$ and $\phi _v$ saturate at a about 4–5%, indicating that there is a maximal amount of particles that can be supported by the air flow. This is in agreement with a critical concentration found in experimental studies on turbulent gas–particle mixtures^6,8^. Above this critical concentration, clusters are formed that can not be maintained in suspension and settle to form a dense fluidized bed at the bottom of the suspension^6^. For a suspension of $77\; \upmu \hbox {m}$ particles the reported value of the critical concentration $\phi _c\sim$ 1%^6^ is lower than the $\sim$ 4% found here.

Good agreement between the experimental data of $\alpha$ and the models is also obtained for suspensions of 155 $\upmu$m glass beads for all the $\phi _i$ tested (Fig. 4c,d). Moreover, for $v_{\theta }$, the ECAH44 model appears able to fit the measured decrease with frequency. However, for higher $\phi _i$ ($>\sim$ 8%) the agreement between velocity data and models fail. This is probably due to noise in the phase measurement, which makes phase unwrapping very challenging at higher $\phi _i$. The locally measured $\phi$’s obtained from the fits have the same features as before, i.e. a linear increase with increasing $\phi _i$ and a apparent saturation $\phi _c\sim$ 9% (Fig. 5a2,b2). For low $\phi _i$, there appears a small difference between the local $\phi 's$ and $\phi _i$ which may be due uncertainties in the determination of the occupied volume -from which $\phi _i$ is calculated- and/or a density gradient in the suspension.

The critical concentrations found here appear higher than those reported in literature^6^. This is most probably due to the fact that here a local solid volume fraction is measured, while in Weit et al. a bulk volume fraction is determined by assuming that the suspension is homogeneous^6,8^. However, as they themselves already point out, there may be a significant density gradient with a denser part at the bottom and a dilute part at the top of the suspension. Furthermore, the shape and size of the container and the corresponding complicated flow may also effect the critical concentration.

It was suggested that the critical concentration in turbulent gas–particle mixtures coincided with the onset of cluster formation by locally enhanced particle concentrations resulting from particle collisions and hydrodynamic instabilities^6,8^. To explore the formation of cluster we acoustically monitor the temporal variations in the locally measured solid volume fraction.

**Figure 6:**
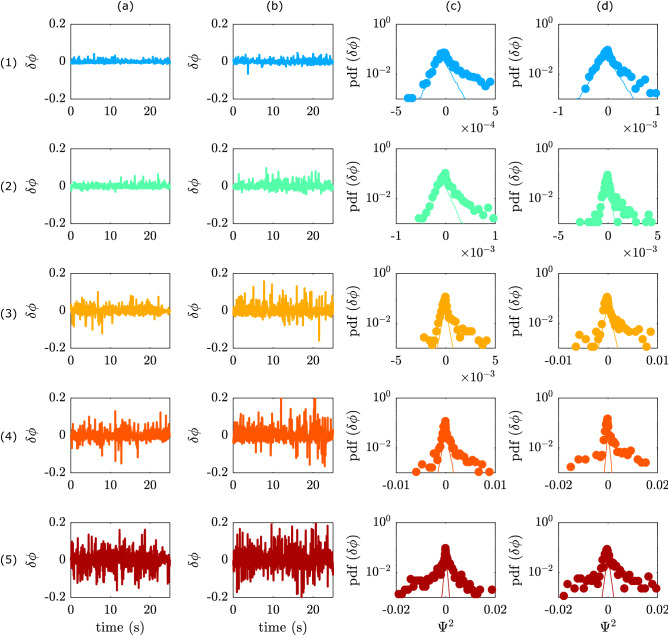
Temporal fluctuations in the local solid volume fraction, obtained from the ultrasound attenuation data, calculated via $\delta \phi (t)=\phi (t)-<\phi >_t$. (**a**) For suspensions of *d* = 77 $\upmu$m particles. Rows 1–5 are $\phi _i$= 0.018, 0.025, 0.032, 0.043 and 0.06. (**b**) For suspensions of *d* = 155 $\upmu$m particles. Rows 1–5 are $\phi _i$= 0.024, 0.036, 0.055, 0.073 and 0.11. (**c**) probability distribution functions (PDF) for the occurrence of fluctuation amplitudes for the traces in (**a**) plotted as a functions of $\Psi ^2$. (**d**) PDF plotted as a functions of $\Psi ^2$ for the traces in (**b**). For comparison Gaussian distributions (which have triangular shape) with variances set to model the left side of the experimental distribution are presented by solid lines in (**c**) and (**d**).

The analysis of the ultrasound attenuation appears most robust, especially at higher $\phi$, therefore we will use only this one in the following analysis. For each experiment and each $\phi _i$ the fluctuations in the $\phi$ are determined (from attenuation) in the course of time during air injection, via: $\delta \phi (t)=\phi (t)-<\phi >_t$, where $<>_t$ denotes the time average. Next, the fluctuations during the experiments for the same $\phi _i$ are pooled. Figure 6a,b, show the fluctuations in $\phi$ for different $\phi _i$. The first observation is that the local $\phi$ is fluctuating around a rather well defined average value, even in suspensions where a dense bed is present at the bottom ($\phi _c>$ 4% for *d* = 77 $\upmu$m). This suggests a steady cycling between the dense bed and the suspension, on the one hand from the suspension to the dense bed via clusters, and, on the other hand, possibly by the ejection of particles from the bed into the suspension as observed in Weit et al.^6^.

To obtain the shape of the distribution function for the fluctuation amplitudes we calculate the normalized probability distribution function (PDF) of $\delta \rho$ and show it as a function of $\Psi ^2=(\phi (t)-<\phi >_t)^2$sgn$(\phi (t)-<\phi >_t)$. The data is divided in 20 equal sized bins. For a Gaussian random process the distribution will have a triangular shape^30^. We find that the PDFs deviate from Gaussian behaviour and develop “fat tails” (Fig. 6c,d). Interestingly, the deviations appear in suspensions below $\phi _c$, and they tend to occur preferentially at positive values of $\Psi ^2$, i.e. higher densities. In analogy, numerical simulations showed that in the inertial range of turbulence (at length scales larger than the Kolmogorov scale) the stationary particle concentration possesses strong fluctuations, which depend on a rescaled contraction rate^31^. It is also worth noting that clustering behavior was observed in dense granular fluids^30^ and granular gasses^32^, in which clustering is driven by dissipating particle–particle collisions. This suggests that clustering mechanisms such as gas–particle interactions and/or particle–particle (collisional) interactions are present in turbulent dilute suspension of dissipating particles even below the critical particle concentration. In line with this argument, the observed increase of clustering for larger particles may be related to either the larger collision section of larger particles and/or the increase of the stokes number for the larger particles. To explain the presence of clusters and the absence of a dense bed in suspensions below the critical particle concentration we hypothesize that cluster lifetime plays an important role. At $\phi < \phi _c$ the size of transient clusters may be small and their lifetime shorter than the time it takes them to drop to the bottom (settling time). Consequently, these clusters disintegrate and the particles are kept in suspension. In contrast, for suspensions at $\phi _c$, the clusters may reach a critical size and become stable over times longer than their settling time, hence, they reach the bottom and form a dense bed. Further studies are necessary to address the size and lifetime of these three dimensional and transient clusters.

To end, we go back to our data on ultrasound propagation at different *U* (Fig. 2). We obtain the $\alpha (f)$ and $v_{\theta }(f)$ from the FFT spectra of the pulses. Since the scattering models yield similar results, the $\phi _\alpha$ and $\phi _v$ are determined from fitting the Ament model, which is more intuitive and faster to implement. We find that at $U^*$ the obtained local $\phi \sim$ 0.007 and 0.008, for *d* = 77 $\upmu$m and *d* = 155 $\upmu$m, respectively, are close to input solid volume fraction $\phi _i=0.006$ (Fig. 2c1,c2). This suggests that all the particles are suspended and confirms that $U^*$ is indeed equal to the settling velocity of the particles. The sharp transition from suspending (nearly) no particles to suspending all particles reflects the (quasi) mono-dispersity of the particles.

## Conclusion

We have introduced acoustic probing to investigate granular suspensions in air. The measured ultrasound attenuation and phase velocity were compared to existing theoretical scattering and hydrodynamic models in order to obtain the solid volume fraction of these dilute suspensions. Using this approach, we confirmed the existence of a maximum local solid volume fraction (critical $\phi$), which depends on the size of the particles. Analysis of the temporal fluctuations in the solid volume fraction indicates that clusters are formed not only above the critical concentration but also below this threshold. This suggests that cluster formation is not directly correlated to the critical concentration, but that other parameters, such as cluster lifetime, may play an important role. We believe that this work can help to improve the fundamental understanding of turbulent fluid-particle mixtures. Furthermore, this relatively straightforward monitoring method on the basis of an intuitive acoustic attenuation model, constitutes an attractive tool to investigate these optically opaque mixtures in analogue laboratory experiments.
